# Evaluation of the *Leptospira interrogans* Outer Membrane Protein OmpL37 as a Vaccine Candidate

**DOI:** 10.1371/journal.pone.0142821

**Published:** 2015-11-20

**Authors:** Thaís Larré Oliveira, André Alex Grassmann, Rodrigo Andrade Schuch, Amilton Clair Pinto Seixas Neto, Marcelo Mendonça, Daiane Drawanz Hartwig, Alan John Alexander McBride, Odir Antônio Dellagostin

**Affiliations:** 1 Programa de Pós-Graduação em Biotecnologia, Centro de Desenvolvimento Tecnológico, Universidade Federal de Pelotas, Pelotas, RS, Brazil; 2 Departamento de Microbiologia e Parasitologia, Instituto de Biologia, Universidade Federal de Pelotas, Pelotas, RS, Brazil; Cornell University, UNITED STATES

## Abstract

The identification of potential vaccine candidates against leptospirosis remains a challenge. However, one such candidate is OmpL37, a potentially surface-exposed antigen that has the highest elastin-binding ability described to date, suggesting that it plays an important role in host colonization. In order to evaluate OmpL37’s ability to induce a protective immune response, prime-boost, DNA and subunit vaccine strategies were tested in the hamster model of lethal leptospirosis. The humoral immune response was evaluated using an indirect ELISA test, and the cytokine profile in whole blood was determined by quantitative real-time PCR. Unlike the DNA vaccine, the administration of recombinant OmpL37 induced a strong IgG antibody response. When individually administrated, both formulations stimulated a TNF-α mediated inflammatory response. However, none of the OmpL37 formulations or vaccination strategies induced protective immunity. Further studies are required towards the identification of new vaccine targets against leptospirosis.

## Introduction

Leptospirosis is a tropical, neglected, zoonotic disease caused by pathogenic spirochetes of the *Leptospira* genus. It is typically associated with inadequate sanitation, poverty, and recreational or professional activities that involve exposure to known risk factors [1–2]. Transmission occurs mainly through direct or indirect contact with the contaminated urine of reservoir animals [3]. The global burden of leptospirosis is estimated to be 890,000 annual cases [4], with approximately 4,000 confirmed cases in Brazil [5]. However, laboratory diagnosis remains a challenge and is unavailable in many countries therefore the actual global prevalence is likely underestimated. Leptospirosis ranges from a mild influenza-like illness to a severe disease that can cause multiple organ failure. The mortality rate varies from 10% (Weil’s disease) to 70% (leptospirosis-associated pulmonary haemorrhage syndrome) [6]. There are approximately 49,000 deaths per year worldwide from Leptospirosis [4].

More than 260 serovars of *Leptospira* spp. have been described, and this antigenic diversity is related to variations in their lipopolysaccharides (LPS) [7]. Commercially available vaccines are bacterin-based (killed whole cells), do not provide cross-protection against different serovars, confer only short-term immunity and usually fail to prevent the transmission of the disease [8–9]. Efforts to develop recombinant vaccines against leptospirosis have focused on conserved outer membrane proteins (OMPs) that represent potential targets for immunological defence mechanisms. Several leptospiral proteins have been evaluated as vaccine candidates; however, to date, no broadly conserved antigen has been able to induce sterilizing and long-term protective immunity. As such, there is a distinct need to characterize new targets [10–11].

OmpL37 is a potential OMP from *L*. *interrogans* and fulfils several requirements for a potential vaccine candidate. It was recently characterized as a cell surface-exposed protein [12], that binds to several extracellular matrix components and has an increased affinity for elastin. Elastin-rich tissues (e.g., skin, lungs, arteries and bladder) are highly relevant to the pathogenesis and transmission of leptospirosis [13]. Furthermore, one study reported that the expression of OmpL37 was up-regulated during infection [14] and within dialysis membrane chambers (DMCs) implanted in rat peritoneum to mimic *in vivo* conditions [15], suggesting that this protein plays an important role in pathogenesis. OmpL37 is highly conserved among pathogenic *Leptospira* spp. and is recognized by patient convalescent sera [13].

DNA and subunit vaccines represent potential intervention strategies for leptospirosis and have been extensively evaluated [16–20]. Both are able to induce a humoral immune response, and DNA vaccines stimulate cell-mediated immunity. The aim of this study was to characterize and evaluate the immunoprotective potential of OmpL37 from *L*. *interrogans* serovar Copenhageni strain Fiocruz L1-130 using prime-boost, DNA, and recombinant protein-based vaccination strategies.

## Materials and Methods

### Bacterial strains and culture conditions

*L*. *interrogans* serogroup Icterohaemorrhagiae serovar Copenhageni strain Fiocruz L1-130 was cultured in Ellinghausen-McCullough-Johnson-Harris (EMJH) liquid medium (Difco; BD, Franklin Lakes, NJ, USA), and was supplemented with *Leptospira* enrichment EMJH (Difco) at 30°C [28]. All procedures with *L*. *interrogans* were conducted using a low passage strain, *i*.*e*. eight passages in hamsters followed by three passages *in vitro*. The *Escherichia coli* strains TOP10 and BL21 (DE3) Star (Invitrogen, São Paulo, Brazil) were grown at 37°C in Luria-Bertani (LB) medium (with the addition of ampicillin to 100 μg.ml^-1^ as required).

### Presence and conservation of *ompL37* in *Leptospira* spp

PCR using the following primers: OmpL37-For (5ˈ-AAGGATCCGATCAGATCAACTTAG) and OmpL37-Rev (5ˈ-TGGGTACCTTAATTTTGTGTTTTTG) flanking the *ompL37* gene (GenBank: 2770109), was performed using genomic DNA from 17 *Leptospira* serovars: *L*. *interrogans* serovars Autumnalis, Bataviae, Bratislava, Canicola, Djasiman, Hebdomadis, Icterohaemorrhagiae, Muenchen, and Pomona, *L*. *borgpetersenii* serovars Ballum, Castellonis, Javanica, Mini, Poi, and Sejroe, *L*. *kirschneri* serovars Cynopteri and Grippotyphosa, and *L*. *santarosai* serovar Pomona. The rRNA 16S gene (positive control) was amplified using the primers fD1_F and rP2_R, as previously described [21]. The OmpL37 protein sequence from *L*. *interrogans* sv. Copenhageni L1-130 (NCBI Reference Sequence: WP_000949351.1) was compared with all the published genome sequences by BlastP in order to determine the conservation level of OmpL37 in different species and serovars; i.e., *L*. *interrogans* sv. Lai strain 56601 [22], *L*. *borgpetersenii* sv. Hardjo strain L550 [23], *L*. *santarosai* sv. Shermani strain LT821 [24], *L*. *licerasiae* sv. Varillal strain VAR010 [25], and *L*. *biflexa* sv. Patoc strain Patoc1 Ames [26]. A BlastP analysis was conducted excluding *Leptospira* genus (taxid: 171) in order to determine the presence of OmpL37 orthologues in other bacteria.

### Cloning of *ompL37*

The *ompL37* PCR product amplified from *L*. *interrogans* sv. Copenhageni strain Fiocruz L1-130 genome, as described above, was cloned into the *E*. *coli* expression vector pAE [27] for expression of an OmpL37 recombinant protein (rOMPL37) with an N-terminal 6×His tag. For construction of the DNA vaccine, *ompL37* was amplified using the primers OmpL37-pTargeT-For (5ˈ-ACCATGGGAGATCAGATCAACTTAG) and OmpL37-Rev and cloned into the mammalian expression vector pTargeT (Promega, Madison, WI, USA). Both plasmid constructs were confirmed by PCR, restriction digestion and DNA sequencing.

### Production of recombinant OmpL37 and Western blot

The recombinant vector pAE/*ompL37* was used to transform *E*. *coli* BL21 Star (DE3) cells (Invitrogen). The 6×His-tagged rOmpL37 protein was expressed, purified by affinity chromatography under denaturing conditions, and characterized by Western blot, as previously described [28]. A pool of convalescent sera collected from severe leptospirosis patients (diluted 1:300) and an anti-human IgG peroxidase conjugate (diluted 1:2,000) were used to confirm that native OmpL37 stimulated the host immune system during infection. The concentration of rOmpL37 was determined using a BCA protein assay kit (Pierce; Thermo Scientific, Rockford, IL, USA). Following this, rOmpL37 (50 μg/dose) was used to produce a mouse anti-rOmpL37 serum, as previously described [29], and the serum was characterized by Western blot.

### *In vitro* expression of rOmpL37 in mammalian cells

CHO-K1 cells were grown on microscope coverslips in six-well plates and transfected with pTargeT/*ompL37* or pTargeT (control), using Nanofect Transfection Reagent (Qiagen) according to the manufacturer’s protocol. Briefly, CHO-K1 cells were maintained in serum-free Dulbecco’s modified Eagle’s medium (DMEM). When 80% confluence was achieved, the CHO-K1 cells were transfected with 2 μg of plasmid DNA mixed with Nanofect reagent in serum-free DMEM. Twenty-four hours after transfection, the functionality and expression of rOmpL37 were evaluated by indirect immunofluorescence assay (IFA) using mouse anti-OmpL37 serum (1:100 in PBS) and anti-mouse IgG-FITC conjugate (1:200 in PBS). CHO-K1 DNA was stained with Hoechst 33258. The reading was performed with a fluorescence microscope at 400× magnification.

### Immunization and challenge of hamsters

Five groups of six female hamsters, aged between 4 and 6 weeks, were immunized in the quadriceps muscle twice, with a 21-day interval between each immunization, as follows: rOmpL37-Alhydrogel (2 × 100 μg), pTargeT/*ompL37* (2 × 100 μg), prime-boost pTargeT/*ompL37* (100 μg) plus rOmpL37 (100 μg), pTargeT (2 × 100 μg) and PBS-Alhydrogel. A positive control group of four hamsters were immunized with 10^9^ heat-killed whole-leptospires. Forty-two days after the first immunization, hamsters were challenged intraperitoneally with a dose of 10^3^ leptospires, equivalent to five times the 50% lethal dose (LD_50_) of the *L*. *interrogans* sv. Copenhageni strain Fiocruz L1-130 [30]. Two independent experiments were conducted. Blood samples were collected from the retro-orbital venous plexus after administration of eye anesthetic drops before each immunization and challenge, and the sera were stored at -20°C. The hamsters were observed daily for end-point criteria, including loss of appetite, gait difficulty, prostration, dyspnea, ruffled fur and weight loss of ≥10% of the animal's maximum weight. The animals that reached end-point criteria and animals that survived through the end of the experiment were humanly euthanized by deep anesthesia using pentobarbital. Survivors were euthanized 30 days post challenge. Following euthanasia, kidney samples were collected for evaluation of leptospiral colonization by *in vitro* culture in EMJH medium, as previously described [28]. All animal procedures were approved by the Ethics Committee in Animal Experimentation of the Federal University of Pelotas, under protocol number 2348.

### Antibody response determination using ELISA

The humoral immune response induced by the different vaccine formulations was evaluated by indirect enzyme-linked immunosorbent assay (ELISA) using rOmpL37 as the antigen. Briefly, rOmpL37 diluted in carbonate-bicarbonate buffer, pH 9.6, was used in a concentration of 50 ng per well. The ELISA plates were washed three times with PBST (PBS with 0.05% [v/v] Tween 20) and blocked with 5% fat-free dry milk. Hamster’s sera were added at a 1:400 dilution in PBS for 1 h at 37°C, and then the plates were washed three times with PBST. Peroxidase-conjugated anti-golden Syrian hamster IgG antibody (Rockland) was added at a 1:6,000 dilution. After incubation at 37°C for 1 h, plates were washed five times with PBST, and the reaction was developed by adding *o*-phenylenediamine dihydrochloride (Sigma-Aldrich) and hydrogen peroxide. The reaction was stopped with 25 μl of 4 N H_2_SO_4_, and the absorbance was read at 492 nm.

### Blood RNA isolation and cDNA synthesis

Total RNA was isolated from pooled blood samples using the RiboPure-Blood Kit (Ambion), according to the manufacturer’s instructions. cDNA synthesis was performed using the High-Capacity cDNA Reverse Transcription Kit (Applied Biosystems) as per the process described by the manufacturer.

### Quantitative real-time PCR assay

Real-time PCR (qRT-PCR) reactions were run on a Stratagene Mx3005P Real-Time PCR System (Agilent Technologies, Santa Clara, CA, USA). The qPCR using SYBR Green PCR Master Mix (Applied Biosystems) and primers (Table 1) was carried out in a 25 μl reaction volume (50 ng cDNA, 12.5 μl Master Mix, 0.5 μM of each primer). The cycling conditions consisted of 95°C for 10 min (denaturation), followed by target DNA amplification for 45 cycles (95°C for 5 s, 60°C or 61°C for 30 s, and a variable extension time at 72°C). The melting curves were analysed immediately after amplification at a linear temperature transition rate of 0.1°C/s from 55 to 95°C, with continuous fluorescence acquisition. The relative C_T_ (ΔΔC_T_) method [31] was used to quantify cytokine gene expression. Briefly, the fold change of each target gene was normalized to the β-actin housekeeping gene CT (ΔC_T_), and compared to a calibrator sample, the same normalized gene in the pre-immune sera sample (ΔΔC_T_). The final value represents the relative fold between immunized and non-immunized hamsters.

**Table 1:** Detailed primers and conditions used for qRT-PCR assays.

Cytokine/Housekeeping Gene	Primer Sequence^a^	Annealing Temp (°C)	*T*_*m*_ (°C)^b^	Amplicon Length (bp)^a^	Nucleotide Sequence Accession Numbers
***TNF-α***	F: AACGGCATGTCTCTCAA	60	50.4	278	AF046215
	R: AGTCGGTCACCTTTCT		49.2		
***IFN-γ***	F: GACAACCAGGCCATCC	60	54.3	226	AF034482
	R: CAAAACAGCACCGACT		49.2		
***TGF-β***	F: ACGGAGAAGAACTGCT	60	42.9	245	AF046214
	R: ACGTAGTACACGATGGG		52.8		
***IL-1α***	F: AGTTCGTCCTGAATGATTCC	61	58.4	202	AB028235
	R: TGGTCTTCACCCTGAGC		59.6		
***β-Actin***	F: TCTACAACGAGCTGCG	60	51.7	357	AJ312092
	R: CAATTTCCCTCTCGGC		51.7		

^a^Vernel-Pauillac and Merien, 2006; Vernel-Pauillac and Goarant, 2010.

^b^Melting temperature.

### Statistical analysis

The Fisher exact test and log-rank test were used to determine significant differences in mortality and survival rates, respectively, among the experimental groups. The Student’s t-test was used to determine significant differences among the serological assays. Differences were considered significant at a *P* value of ≤ 0.05. The analyses were carried out with GraphPad Prism 4 and QuickCalcs software.

## Results

### Distribution of *ompL37* among *Leptospira* spp

PCR analysis showed that the *ompL37* gene is present in *L*. *interrogans* (nine serovars), *L*. *borgpetersenii* (six serovars), *L*. *kirschneri* (two serovars), and *L*. *santarosai* (one serovar). *In silico* protein sequence analysis showed that the predicted OmpL37 sequence in *L*. *interrogans* sv. Copenhageni was 100% identical to the LA1495 sequence in *L*. *interrogans* sv. Lai. Compared to OmpL37 in *L*. *borgpetersenii* sv. Hardjo, *L*. *santarosai* sv. Shermani and *L*. *licerasiae* sv. Varillal, the identity was 88, 87 and 63% with a query coverage of 100%, 100% and 99%, respectively. A less conserved protein orthologue was observed in saprophytic *L*. *biflexa* with an identity of 46% (94% coverage). Outside *Leptospira* genus, proteins with 32% and 28% of identity, with over 90% of coverage, were identified in spirochetes *Turneriella parva* and *Leptonema illini* (Leptospiraceae), respectively.

### Subunit and DNA vaccine preparation

**Fig 1:**
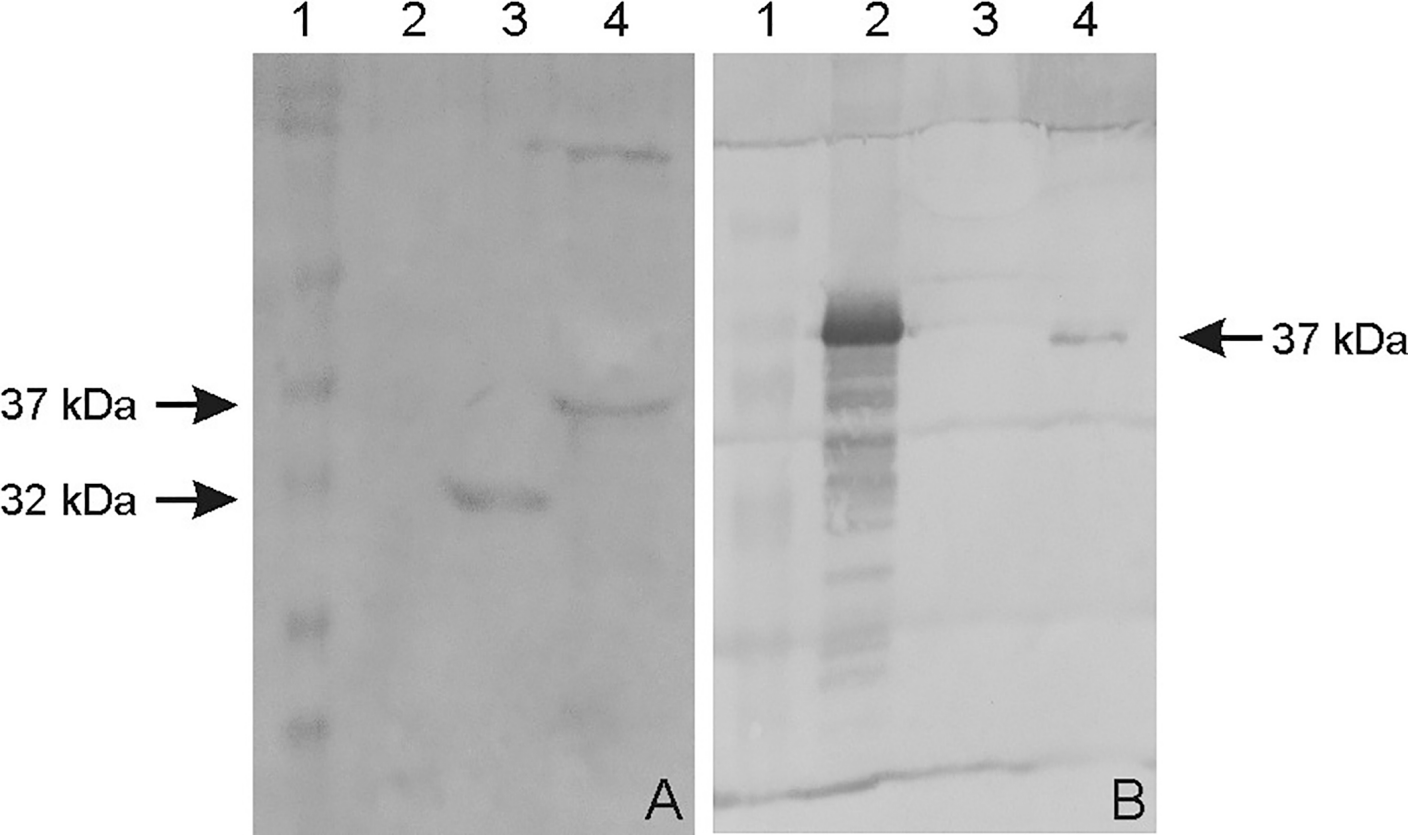
Western blot of recombinant and native OmpL37 proteins. A: rOmpL37 characterization with convalescent human sera. (1) Full-Range Rainbow Molecular Weight Marker (GE Healthcare); (2) Negative control (BSA); (3) Positive control (rLipL32); (4) rOmpL37. B: Anti-rOmpL37 serum characterization. (1) Full-Range Rainbow Molecular Weight Marker (GE Healthcare); (2) rOmpL37; (3) Negative control (BSA); (4) *L*. *interrogans* serovar Copenhageni Fiocruz L1-130 WCL.

**Fig 2:**
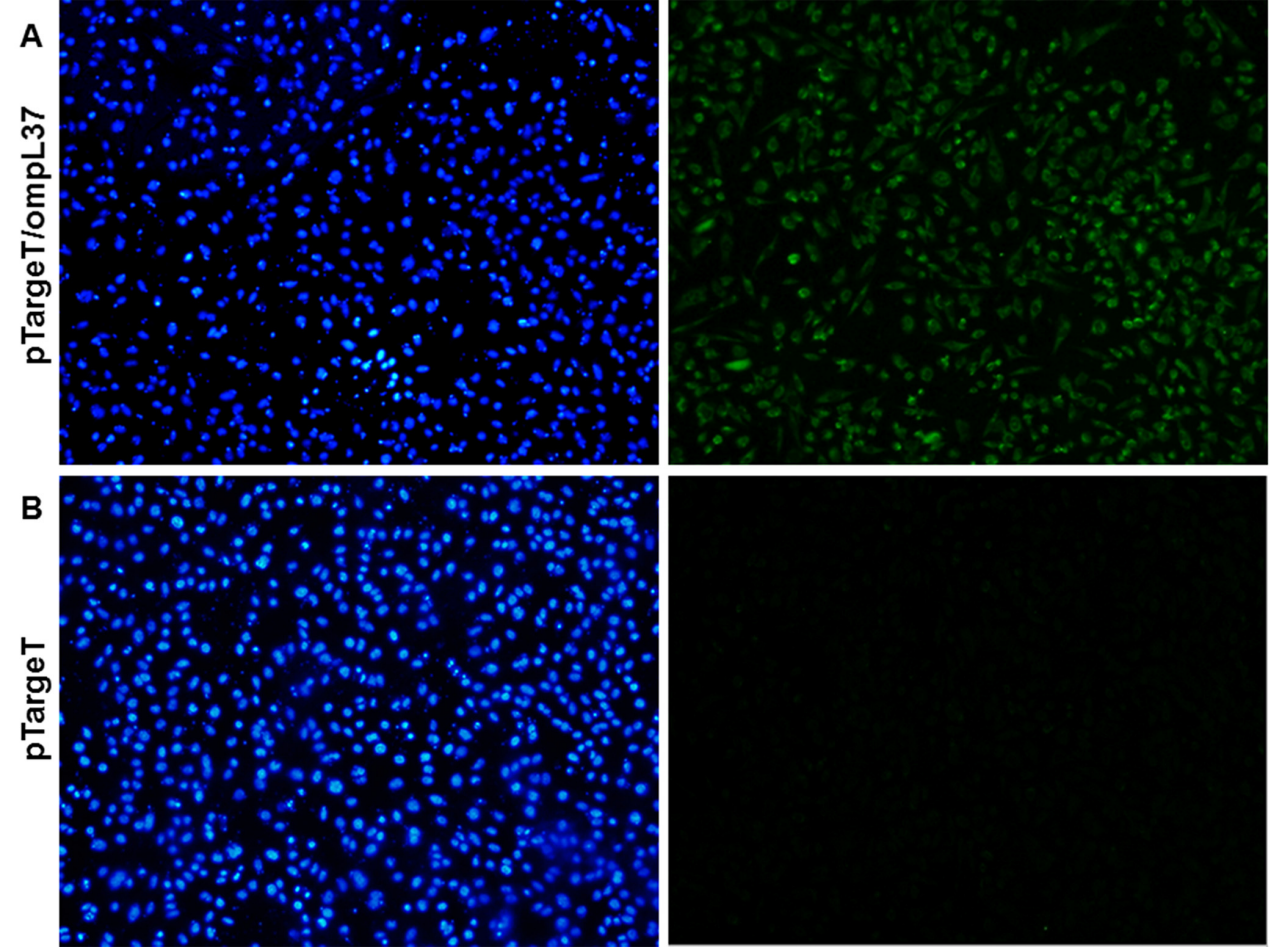
Immunofluorescence (IFA) analysis of the expression of recombinant OmpL37 protein in CHO-K1 cells 24 h after transfection with pTargeT/*ompL37* (A) or pTargeT alone (B). The IFA was based on a mouse anti-rOmpL37 antibody and FITC conjugated anti-mouse IgG. Panels on the left shows Hoechst 33258 DNA staining and on the right, antibody reactions.

The *ompL37* plasmid sequences were confirmed by sequencing. The OmpL37 recombinant protein was expressed in inclusion bodies in *E*. *coli* with the expected size of 37 kDa. After dialysis against PBS, the protocol for solubilization in urea and purification of the recombinant protein resulted in a yield of 10.4 mg.L^-1^. The rOmpL37 was recognized by convalescent sera evaluated by Western blot. Mouse anti-rOmpL37 serum recognized the native OmpL37 protein in *L*. *interrogans* sv. Copenhageni strain Fiocruz L1-130 whole cell lysate (WCL), Fig 1. The expression of OmpL37 in the pTargeT construct was confirmed through the detection of rOmpL37 in transfected CHO-K1 cells (Fig 2).

### Humoral immune response in vaccinated hamsters

**Fig 3:**
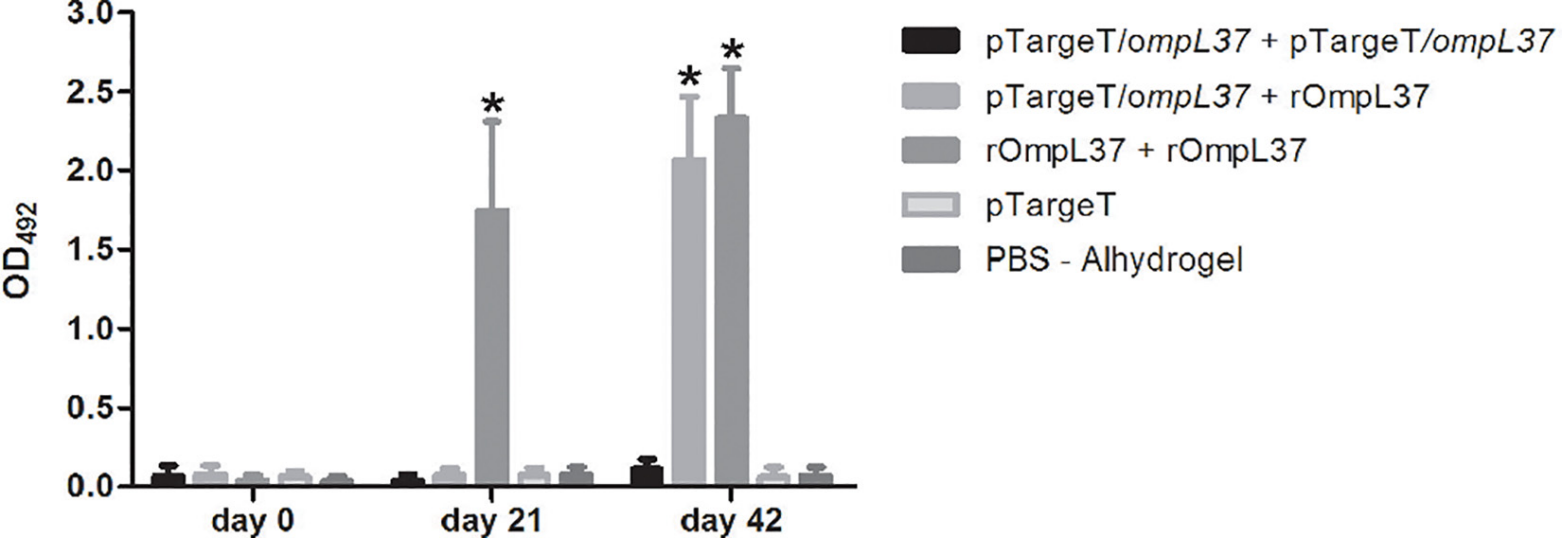
Specific IgG response in hamsters inoculated with different vaccine formulations. Recombinant rOmpL37 expressed by *E*. *coli* was used as the antigen in ELISA. Mean values were calculated from serum samples assayed in triplicate. Results are expressed as the mean absorbance ± standard deviation. OD_492_, optical density at 492 nm. Significant differences, at a *P* value of 0.001 in comparison to the control group, are shown by an asterisk.

In order to assess the specific antibody response in groups of hamsters immunized with the OmpL37 vaccine preparations, an indirect ELISA was performed with the sera from each animal collected on days 0 (pre-immune), 21 and 42 post-immunization (pi), using rOmpL37 as the immobilized antigen (Fig 3). The rOmpL37 vaccine induced significantly higher antibody levels than the control group (*P* < 0.05). A significant response was observed in the prime-boost group following the rOmpL37 boost on Day 42, while the DNA vaccine failed to induce a significant immune response.

### Cytokine expression profile in hamsters immunized with OmpL37

**Fig 4:**
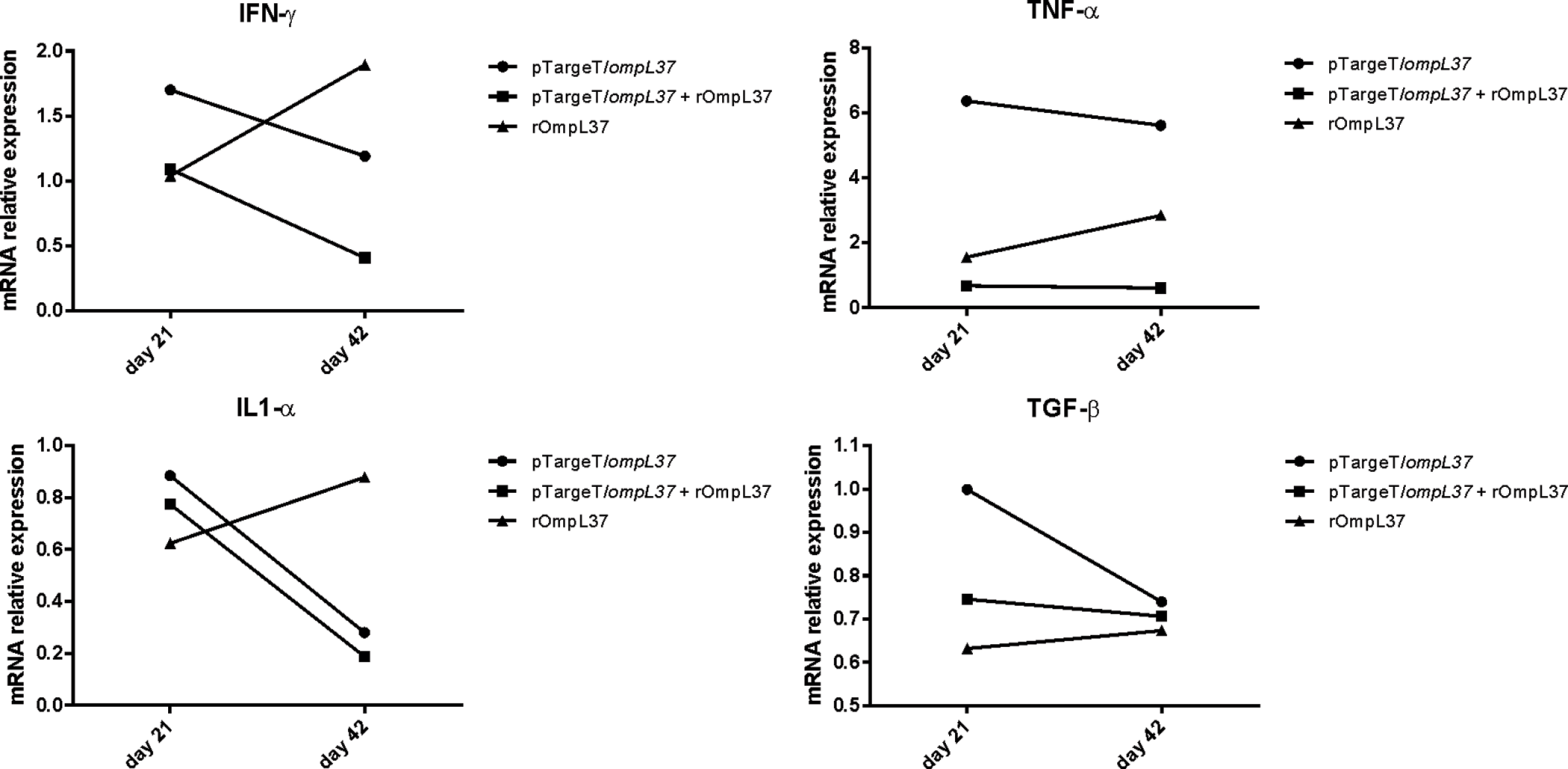
Relative mRNA expression levels of IFN-γ, TNF-α, IL1-α and TGF-β in pooled hamster blood samples. The relative C_T_ (ΔΔ C_T_) method was used to quantify cytokine gene expression: C_T_s were normalized against the β-actin gene C_T_ (Δ C_T_) and then compared to the same normalized gene in the pre-immune sera sample (calibrator). A 2-fold or greater change in mRNA levels was considered significant. The control groups were set to 1. The values represent grouped results of two independent experiments.

The induction of IFN-γ, IL-1α, TNF-α and TGF-β was evaluated by qRT-PCR and only a 2-fold or greater change in mRNA levels was considered significant [32]. IFN-γ (ratio = 0.41) and IL-1α (ratio = 0.19) mRNA expression levels were down-regulated in the prime-boost group at Day 42. In contrast, TNF-α expression increased at Day 42 in animals immunized with rOmpL37 (ratio = 2.84). Following immunization with the DNA vaccine, TNF-α was up-regulated at Days 21 (ratio = 6.4) and 42 (ratio = 5.6), while IL-1α (ratio = 0.28) was down-regulated at Day 42. TGF-β was expressed at basal levels in the vaccinated hamsters (Fig 4).

### Efficacy of the OmpL37 vaccine preparations

**Fig 5:**
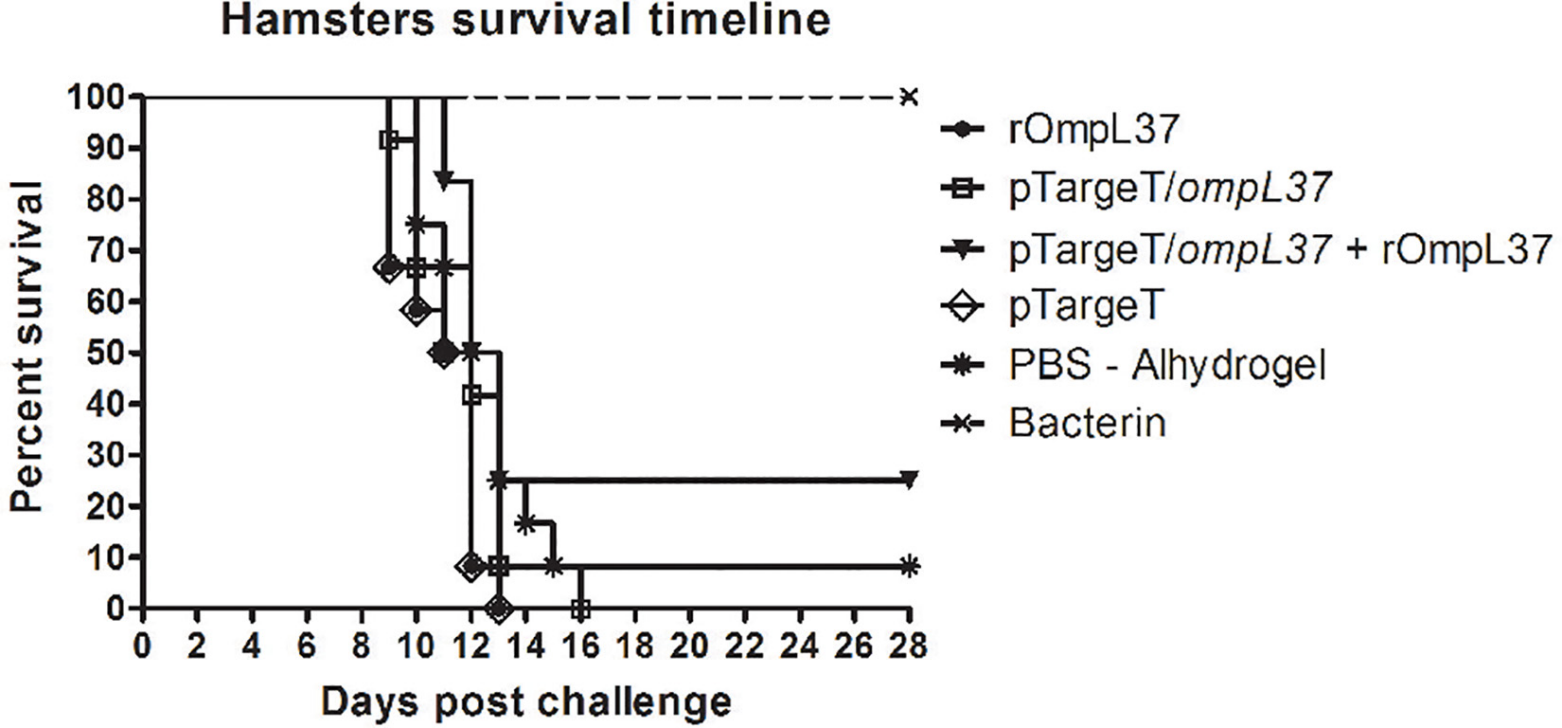
Survival of hamsters immunized with rOmpL37 vaccines after lethal challenge. Survival curves were compared using log-rank analysis. The results are a summary of two independent experiments (Table 2).

The protective efficacy of the OmpL37 vaccine preparations was determined in two independent experiments. The statistical analyses are presented in Table 2, and the survival rates are shown in Fig 5. Considering both protection against mortality and increased survival, none of the OmpL37 vaccine formulations induced a protective immune response. The maximum efficacy of 25% was observed in the prime-boost group. In addition, leptospires were present in the kidneys of the surviving animals, including those in the bacterin group, indicating a lack of sterilizing immunity.

**Table 2:** Effect of immunization with OmpL37 vaccines in hamsters.

Treatment Group	No. Survivors/Total (% protection)			% Culture
	#Exp 1	#Exp 2	#Total	Positive
**rOmpL37**	0/6 (0)	0/6 (0)	0/12	ND
**pTargeT/*ompL37***	0/6 (0)	0/6 (0)	0/12	ND
**pTargeT/*ompL37* + rOmpL37**	3/6 (50)	0/6 (0)	3/12 (25)	3/3 (100%)
**pTargeT**	0/6 (0)	0/6 (0)	0/12	ND
**PBS-Alhydrogel**	1/6 (16.6)	0/6 (0)	1/12 (8.3)	1/1 (100%)
**Killed-whole leptospires**	4/4 (100)	4/4 (100)	8/8 (100)	4/8 (50%)

## Discussion

Traditional bacterin vaccine preparations used in humans and animals present several limitations including severe side effects, short-term immunity and restricted-serovar protection. Several studies have evaluated different formulations of recombinant vaccine candidates with the intention of improving leptospirosis vaccines. To date, the most promising results were obtained using the Lig (Leptospiral immunoglobulin-like) proteins. Mice immunized with LigA or LigB survived lethal challenge, showing 90–100% of protection [33]. A vaccine using the C-terminal portion of the LigA protein, induced protection ranging from 63% to 100% in hamsters [28]. Of note, sterilizing immunity has not yet been achieved [11, 34] and LigA is not present in *Leptospira* spp., highlighting the need for new conserved antigens for vaccine development. This study evaluated, for the first time, the outer membrane protein OmpL37 from *L*. *interrogans* sv. Copenhageni strain Fiocruz L1-130 as a vaccine antigen against leptospirosis. In addition to the localization of OmpL37 on the surface of the outer membrane of the leptospiral cell and a possible role during infection, orthologues of the *ompL37* gene were present in different serovars, suggesting it is a potential candidate for a cross-protective vaccine [12–14].

One limitation that hinders the development of vaccines against leptospirosis is the lack of correlates of protection, immune markers that have contributed to many vaccinology studies [35–36]. To date, no markers have been identified for leptospirosis and the hamster model of lethal leptospirosis remains the preferred method of assessing vaccine efficacy [37–38]. Both humoral and cellular immune responses have been reported to play roles in the response against leptospirosis [39]; as such, this study included a protein-boost strategy that aimed to improve the immunogenicity of the OmpL37 DNA vaccine, as previously demonstrated for other antigens [18–19]. Independent experiments using subunit, DNA vaccine, and prime-boost immunization strategies were performed. A non-protective humoral immune response was stimulated by rOmpL37 (Fig 2) and survival among animals in the prime-boost group was not associated with increased IgG levels (data not shown). Although the functionality of the DNA vaccine was confirmed by the transfection assay, it did not induce an IgG response and this was in agreement with previous observations [19].

Due to the lack of reagents to measure serum concentrations of Syrian hamster immune effectors, studies have been limited to determining cytokine profiles at the transcriptional level [32, 40]. This analysis revealed increased circulating TNF-α in hamsters vaccinated with rOmpL37 (Day 42) or the DNA vaccine (Days 21 and 42) compared to the control groups. Delayed and sustained TNF-α production has been associated with a poor prognosis during infection [41]. High levels of IL1-α seems to be related to severe disease [32]. In our experiments, IL1-α was down-regulated in the OmpL37 prime-boost and the DNA vaccine groups. Previous research has indicated that levels of TNF-α and IL1-α are higher in hamsters infected with virulent leptospires than those infected with an avirulent strain [32]. IFN-γ production was related to protection in cattle vaccinated with monovalent serovar Hardjo vaccines [42]. In the present study, however, increased IFN-γ mRNA levels were found in animals vaccinated with rOmpL37, and this vaccine preparation did not protect against lethal infection. The role of the cytokine profile in protecting against leptospirosis remains poorly understood and needs further investigation.

*E*. *coli*-based expression systems are widely used, despite the fact that they are unable to carry out post-translational modifications such as glycosylation, methylation and acetylation [43]. This is usually irrelevant for bacterial diseases vaccines; however, there is evidence that pathogenic *Leptospira* spp. have protein modification systems [44]. Furthermore, lipidation improved the immunogenicity of recombinant antigens, such as OspA from *Borrelia burgdorferi* and LigANI from *L*. *interrogans* [45–46]. Altered immunogenic properties may have affected the folding of rOmpL37.

Although three animals survived in the prime-boost group, none of the OmpL37 vaccine formulations evaluated in this study induced significant protection against lethal leptospirosis. Even though it is a recurring issue in the animal models of leptospirosis [47–48], this was somewhat unexpected due to the many attractive features inherent in OmpL37, including the induction of immune responses following vaccination, as shown here. Any attempt to explain the lack of protection in this study would be highly speculative. Further experiments are necessary to confirm the distribution of OmpL37 across the outer membrane, the exposed regions and any redundancy of function. Once distribution across the outer membrane is confirmed, additional studies employing different approaches, including alternative adjuvants and vaccine immunization strategies, would be viable. Our group is currently applying different rational approaches, ranging from bioinformatics to serological investigation of leptospiral surface exposed OMPs, to identify new antigens for the development of an effective vaccine against leptospirosis.

## Conclusions

OmpL37, potentially a surface-exposed outer membrane protein expressed during host infection, induced strong immune responses, but failed to stimulate protection against leptospirosis when presented as a recombinant protein in Alhydrogel and DNA vaccine preparations. Further studies are necessary to identify new potential vaccine candidates against lethal leptospirosis.
